# Systematic Review: Adipose-Derived Mesenchymal Stem Cells, Platelet-Rich Plasma and Biomaterials as New Regenerative Strategies in Chronic Skin Wounds and Soft Tissue Defects

**DOI:** 10.3390/ijms22041538

**Published:** 2021-02-03

**Authors:** Pietro Gentile, Simone Garcovich

**Affiliations:** 1 Department of Surgical Science, Plastic and Reconstructive Surgery, “Tor Vergata” University, 00133 Rome, Italy; 2 Scientific Director of Academy of International Regenerative Medicine & Surgery Societies (AIRMESS), 1201 Geneva, Switzerland; 3 Institute of Dermatology, F. Policlinico Gemelli IRCSS, Università Cattolica del Sacro Cuore, 00168 Rome, Italy; simgarko@yahoo.it

**Keywords:** adipose-derived mesenchymal stem cells (AD-MSCs), platelet-rich plasma (PRP), biomaterials, plastic surgery, regenerative plastic surgery

## Abstract

The number of clinical trials evaluating adipose-derived mesenchymal stem cells (AD-MSCs), platelet-rich plasma (PRP), and biomaterials efficacy in regenerative plastic surgery has exponentially increased during the last ten years. AD-MSCs are easily accessible from various fat depots and show intrinsic plasticity in giving rise to cell types involved in wound healing and angiogenesis. AD-MSCs have been used in the treatment of soft tissue defects and chronic wounds, employed in conjunction with a fat grafting technique or with dermal substitute scaffolds and platelet-rich plasma. In this systematic review, an overview of the current knowledge on this topic has been provided, based on existing studies and the authors’ experience. A multistep search of the PubMed, MEDLINE, Embase, PreMEDLINE, Ebase, CINAHL, PsycINFO, Clinicaltrials.gov, Scopus database, and Cochrane databases has been performed to identify papers on AD-MSCs, PRP, and biomaterials used in soft tissue defects and chronic wounds. Of the 2136 articles initially identified, 422 articles focusing on regenerative strategies in wound healing were selected and, consequently, only 278 articles apparently related to AD-MSC, PRP, and biomaterials were initially assessed for eligibility. Of these, 85 articles were excluded as pre-clinical, experimental, and in vitro studies. For the above-mentioned reasons, 193 articles were selected; of this amount, 121 letters, expert opinions, commentary, and editorials were removed. The remaining 72 articles, strictly regarding the use of AD-MSCs, PRP, and biomaterials in chronic skin wounds and soft tissue defects, were analyzed. The studies included had to match predetermined criteria according to the patients, intervention, comparator, outcomes, and study design (PICOS) approach. The information analyzed highlights the safety and efficacy of AD-MSCs, PRP, and biomaterials on soft tissue defects and chronic wounds, without major side effects.

## 1. Introduction

A scientific, clinical need exists for the development of biotechnologies to improve wound healing (WH), soft tissue defects (STDs), and skin repair (SR) in regenerative plastic surgery (RPS).

The number of investigations evaluating the efficacy of autologous platelet-rich plasma (PRP), adult stem cell-based therapy (A-SC-BT), in particular those based on adipose-derived mesenchymal stem cells (AD-MSCs) and biomaterials, have exponentially increased during the last decade (2010–2020).

As the largest organ of the body, the skin (consisting of the epidermis, the dermis, and its appendices), acts as an important barrier against the invasion of foreign microorganisms and has the functions of immunity, thermoregulation, and metabolic activities [1]. Skin defects resulting from burns, chronic diseases, trauma, tumor resection, and so forth often cause water-electrolyte imbalances and microbial invasion, threatening people’s lives.

Autografts, particularly split-thickness skin graft (STSG) or skin flap transplantation, are considered to be important managements for aiding full-thickness skin defects [2,3]. Due to the disadvantages (e.g., complicated operation, severe damage to the donor area, bloated appearance, high failure rate, etc.), skin flap transplantation is not as widely used in clinical practice as skin grafts [4]. However, despite the better take-in in the early stage, skin grafts lacking sufficient dermal matrix are often hindered by uncontrollable scar hyperplasia, lower mechanical resistance, and so forth in the later phase, leading to graft failure or severe scar formation, which seriously affects the local appearance and functions [5]. Understanding how to avoid severe scar hyperplasia and contracture in the later phase is a key difficulty that needs to be overcome in skin grafting. Whether the skin graft has sufficient blood supply is the main factor affecting the quality of the skin graft [6].

PRP is a high-concentration platelet-oriented plasma obtained from animal or human whole blood via centrifugation [7], which is typically 3- to 7-fold of the mean platelet concentration in whole blood. Containing α-granules, platelets secrete several growth factors (GFs) after being activated, such as transforming growth factor-β (TGF-β), platelet-derived growth factor (PDGF), vascular endothelial growth factor (VEGF), and so forth [8,9]. These GFs and other proteins (such as adhesion molecules and chemokines) interact with the local environment to promote cell differentiation and proliferation, which are responsible for re-epithelization and angiogenesis via mesenchymal cell recruitment and extracellular matrix synthesis [8,10]. Nevertheless, PRP is limited due to burst release and its short half-life; that is, 95% of these GFs are secreted within an hour, quickly dilute, and decay into the tissue fluid [11,12]. In this case, although secreted GFs promote angiogenesis and fibroblast maturation, this release process will inevitably cause extensive waste of GFs. In practice, knowing how to avoid the burst release and reduce the waste is a hot issue that needs to be addressed during the use process.

In the past few decades, numerous bi-layer dermal substitutes have been developed and applied for the management of full-thickness skin defects, such as Alloderm^®^ [13,14], Integra^®^ [15,16], Pelnac^®^ [17], and Lando^®^, of which the low-layer porous collagen sponge scaffold (CSS) function as a dermal regenerate template (DRT). Being the main protein of the extracellular matrix, collagen has excellent biocompatibility with low immunogenicity [18]. Moreover, collagen scaffolds have attracted the most attention due to their sustained drug release properties [19]. Previous studies have demonstrated that the CSSs have surfaces of high porous structure that can be functionalized to provide a biomimetic three-dimensional microenvironment, possessing the ability to host drug molecules to sustain drug release [20]. On the other hand, the porous structure guides inward proliferation and the migration of fibroblasts and endothelial cells, which promotes granulation tissue and angiogenesis for wound healing with less scarring [21,22]. However, the second-step skin graft surgery is usually performed two to three weeks after CSS implantation [23], which increases the patient’s pain and prolongs the patient’s hospital stay. One-step skin grafting requires the dermal substitute and a split-thickness skin graft to be covered on the wound at the same time. The existence of a non-vascular active dermal substitute may hinder the survival of the graft [24]. It is known that functional surface coatings mediate the cell-surface interaction between the tissues and the biomaterials, which provide alternative strategies for the improvement of bioactivities [25,26].

In this field, the aim of regenerative strategies must be the development of new autologous biotechnologies to promote WH by ex vivo and in vitro culture, or by in vivo regeneration and bio-stimulation. Autologous A-SC-BT has been of great interest for application in WH and SR. Some early efforts in the field focused on isolating primary cells from a biopsy of the tissue of interest and growing the cells ex vivo for subsequent introduction back into the patient.

The preliminary outcomes related to the use of a new regenerative technique to provide autologous A-SC-BT involving human AD-MSCs to be used in patients affected by STDs and chronic skin wounds (CSW) have been reported [27,28,29,30,31,32,33,34,35,36,37,38,39,40,41,42,43,44,45,46,47,48,49,50,51]. The AD-MSCs were obtained by multiple procedures of centrifugation, filtration, and/or fragmentation [51]. However, a major limitation encountered in this area has been the difficulty in expanding cells to sufficient numbers for human use, the necessity to perform this expansion in good manufacturing practices (GMP) laboratories, and the viability of the expanded cells [51]. For this reason, the clinical use of A-SC-BT to improve WH and STDs has not been adequately considered.

Alternatively, the use of autologous platelet-derived growth factors, contained in PRP, may represent a valid regenerative strategy for their capacity to promote cell proliferation, differentiation, and neo-angiogenesis, favoring, in vivo, the WH process [52], and hair regrowth (HR) has been reported recently [53,54,55,56,57,58].

In this systematic review, data from investigations reporting the use of AD-MSCs, PRP, and biomaterials in CSW and STD treatments to evaluate such interventions’ efficacy, were analyzed.

## 2. Methods

### 2.1. Search Strategy and Literature Screening

The research was conducted in accordance with the PRISMA guidelines and the Cochrane handbook [59]. A multistep search of the PubMed, MEDLINE, Embase, PreMEDLINE, Ebase, CINAHL, PsycINFO, Clinicaltrials.gov, Scopus, and Cochrane databases was performed to identify studies, published before November 1, 2020, on CSW, STDs, and SR treatment with PRP, AD-MSCs, and biomaterials, searching without a language or publishing-time restriction.

**Figure 1:**
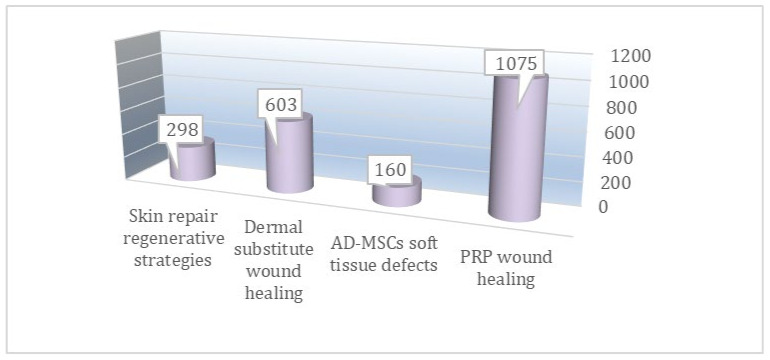
Papers initially found on autologous AD-MSCs, PRP, and biomaterial applications in soft tissue defects and chronic skin wounds.

There were 1075 articles that used the keyword “PRP wound healing”, 160 articles that used the keyword “adipose-derived mesenchymal stem cells soft tissue defects”, 603 that used the keyword “dermal substitute wound healing”, and 298 that used the keyword “skin repair regenerative strategies” found, as shown in Figure 1. The articles related to “PRP wound healing” *(n* = 1075), “adipose-derived mesenchymal stem cells soft tissue defects” *(n* = 160), “dermal substitute wound healing” *(n* = 603), and “skin repair regenerative strategies *(n* = 298)” were contained in the total initial amount *(n* = 2136).

### 2.2. Study Assessment

The aim of this systematic review was to assess the selected articles comparing these regenerative strategies (PRP, AD-MSCs, and biomaterials) compared to any control for WH and SR. Articles included in this work had to match predetermined criteria according to the patients, intervention, comparator, outcomes, and study design (PICOS) approach (https://ro.ecu.edu.au/cgi/viewcontent.cgi?referer=https://www.google.it/&httpsredir=1&article=1010&context=ecupres). The study assessment was based on inclusion and exclusion criteria.

Inclusion criteria:

**P**—Patients (ages 18–79 years, who suffered CSW and STDs);

**I**—Intervention (local application of autologous PRP, AD-MSCs, and biomaterials as a dermal substitute, and hyaluronic acid);

**C**—Comparator (any type of control, internal, external, and different product);

**O**—Outcomes (WH, SR, and soft tissue volume maintenance);

**S**—Study design (clinical trial, randomized placebo-controlled trial/randomized, double-blind, placebo- and active-controlled, half-head study/double-blind, placebo-controlled pilot study/blinded, randomized clinical trial).

Exclusion Criteria:

**P**—Patients (other types of wounds as bone wounds or cartilage wounds); 

**I**—Intervention (advanced dressing, drugs, expanded cells suspensions);

**C**—Comparator (not applied);

**O**—Outcomes (not applied);

**S**—Study design (expert opinion, comments, letter to the editor, single case report, preclinical model, animal studies, in vitro studies, articles identified as bias, not correct match with the keyword used, shorter follow up than three months). No limitations were applied on ethnicity or method of PRP/AD-MSCs processing.

This systemic review, performed with the PICOS approach, is considered an evidence-based medicine (EBM) Level 1a study, according to the Oxford Centre for Evidence-Based Medicine (OCEBM), March 2009 (https://www.cebm.net/2009/06/oxford-centre-evidence-based-medicine-levels-evidence-march-2009/).

### 2.3. Study Selection

Original studies including the research article, observational studies (i.e., case series, cross-sectional, case-control, and cohort), and randomized trials in Italian, English, German, Swedish, Norwegian, Spanish, Danish, Turkish, American, and Chinese were all eligible for inclusion.

Exclusion criteria were studies only including abstracts, unpublished studies, and a lack of raw data. Conference reports were also excluded for insufficient details for analysis. The titles and abstracts of the identified studies were performed by the two investigators (P.G. and S.G.). If the information provided in the abstracts was not sufficient to access the eligibility, a full-text evaluation was conducted. The two authors (P.G. and S.G.) also evaluated the quality of the included studies independently. Any disagreement was resolved through discussion.

Of the 1075 articles, 197 using the keyword “PRP wound healing" were strictly focused on “PRP healing chronic wound”; 48 of the 160 articles related to “adipose-derived mesenchymal stem cells soft tissue defects” (bias n = 112) were strictly focused on AD-MSCs in wound healing; 109 of the 603 articles related to “dermal substitute wound healing” were strictly focused on “dermal substitute chronic wound healing”; and 68 of the 298 articles related to “skin repair regenerative strategies” *(n* = 298) were strictly focused on “skin repair regenerative strategies in vivo”.

**Scheme 1:**
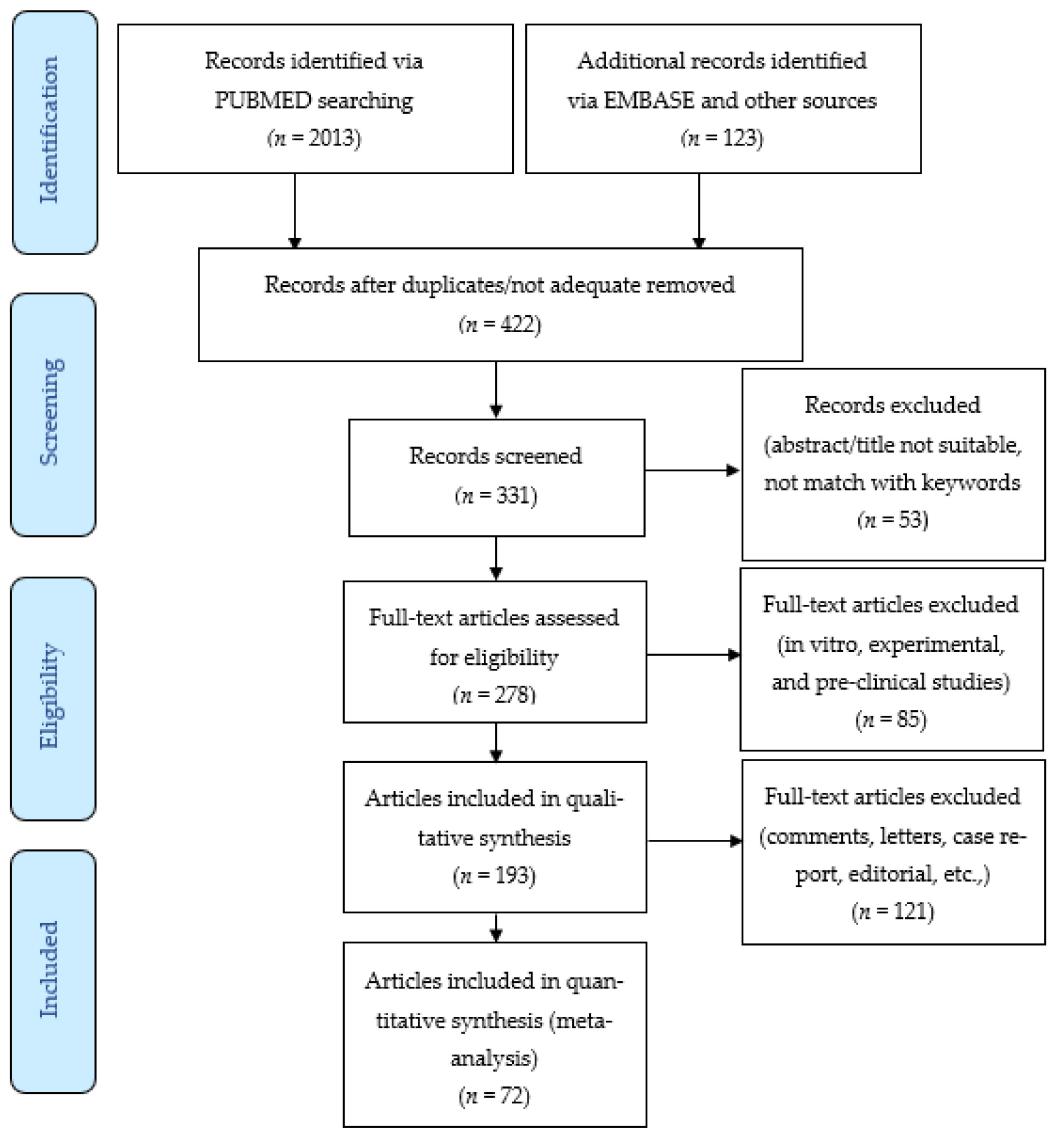
PRISMA (Preferred Reporting Items for Systematic Review and Meta-analysis) flow diagram.

In total, 422 articles focusing on regenerative strategies (RS) in WH based on PRP, AD-MSCs, and biomaterials were initially identified and selected using a Preferred Reporting Items for Systematic Review and Meta-Analysis (PRISMA) flow diagram (www.prisma-statement.org) (Scheme 1).

A total of 1714 articles were excluded as duplicates and/or not adequate. Consequently, it was decided to include only clinical trials with patients diagnosed with CSW, diabetic foot ulcers, vascular and post-traumatic ulcers, soft tissue defects, or skin defects.

### 2.4. Data Extraction

Data were independently extracted by the first investigator (P.G.) and checked by the second investigator (S.G.) only from the retrieved articles. Any disagreement on the extracted data was settled by consensus between P.G. and S.G. No attempt was made to obtain specific or missing data from the authors. The following data were extracted: first author, year of publication, study design, number of patients, type of procedure, and primary and secondary outcomes.

The quality of the included investigations was independently assessed by two investigators (P.G. and S.G.) using the Cochrane Collaboration’s Risk of Bias Assessment tool for Randomized Clinical Trials (RCTs) [59], and the Newcastle–Ottawa Scale to evaluate the individual non-randomized studies [60].

### 2.5. Endpoint Definition

The efficacy of PRP, AD-MSCs, and biomaterials was primarily evaluated by an increase in WH and SR, and secondarily, by an increment of soft tissue volume (STV), satisfaction of patients from the surveys, and changes in STV compared with pictures and Magnetic Resonance Imaging (MRI) analysis taken before and after the treatment sections. Given that various test methods were taken through the studies we included, only the most widely used methods would be set at the endpoints for all pooled studies. All side effects, including local injection pain, increasing sensitivity, biomaterial rejection, and any allergic effects, were analyzed.

## 3. Results

### 3.1. Literature Search

A total of 422 articles were initially identified and 1714 articles were excluded for several reasons, including duplicates *n* = 333), not a correct match after the title’s/abstract’s screening *n* = 301), not human studies *n*= 276), not related to RS *n* = 164), not related to PRP, AD-MSCs, and biomaterials *n* = 442), and not correct match with the topic after full-text reading *n* = 198).

There were 91 articles that were considered bias (not a correct match with the treatments), while 53 articles were excluded due to the abstract/title not being suitable or not matching with the keywords used).

There were 278 articles that were initially assessed for eligibility; of this amount, 85 articles were excluded as pre-clinical, experimental, and in vitro studies. For the above-mentioned reasons, 193 articles were selected; of this amount, 121 articles were letters, expert opinion, commentary, and systematic reviews, and were removed. The 72 articles that strictly related to the use of RS based on AD-MSCs, PRP, and biomaterials in CSW and STDs were analyzed (Scheme 1).

### 3.2. Study Subjects

Among all 422 studies, only 72 studies were found to be relevant. The mean age of the total enrolled patients was above 38 years old. Most patients had a history of CSW, diabetic foot, or skin defects for at least 1–2 years. Laboratory tests, including hemoglobin, platelet count, serum ferritin, liver function, thyroid function, and infection diseases (HBV, HCV, and HIV) were checked for excluding any other autoimmune or systematic diseases.

### 3.3. Platelet-Rich Plasma Preparation

Different methods of PRP preparation were found in the analyzed articles. In detail, several commercial PRP kits, products from different companies, were commonly used, associated with different centrifugation protocols. The choices of activators and anticoagulation varied depending on PRP kits and study purposes. In all enrolled studies, calcium gluconate at a 10:1 ratio and sodium citrate were mostly added as activators and anticoagulants, respectively. Moreover, platelet concentrations differed from 1.5 to 7 times as much as the whole blood, according to PRP preparation protocols [52,58].

### 3.4. Adipose-Derived Mesenchymal Stem Cells Preparation

Two different kinds of AD-MSCs isolation and selection procedures have been commonly described as "enzymatic digestion" vs "non-enzymatic digestion".

Enzymatic digestion of fat tissue is the most used isolation method to obtain AD-MSCs. Typically, collagenase, dispase, or trypsin are used for the digestion of fat tissue [50]. Even though the techniques for the isolation of said cells are fairly diverse, they still stick to a standard process. Frequently used collagenases are type I and type II [50]. The plastic-adherent cell fraction, including AD-MSCs, can be obtained after passaging or cryopreservation, or further cultivated for expansion for a more homogeneous AD-MSC population. As the application of enzymes is characterized by high costs and might have an impact on safety and efficacy [50], several researchers have focused on "non-enzymatic procedures", using centrifugation, filtration, and micro-fragmentation. Mechanical procedures like centrifugation, filtration, and micro-fragmentation have replaced the enzymatic digestion to isolate AD-MSCs.

### 3.5. Biomaterial Application

Skin is the largest organ of the body with many essential functions. Due to its direct contact with the external environment, which makes it extremely prone to damage and/or injury, the skin plays a crucial role as a barrier against exogenous substances, pathogens, and mechanical stresses.

In the past few decades, numerous bi-layer dermal substitutes have been developed and applied for the management of full-thickness skin defects, such as Alloderm^®^ [13,14], Integra^®^ [15,16], Pelnac^®^ [17] and Lando^®^, of which, the low-layer porous CSS function as DRT. Being the main protein of the extracellular matrix, collagen has excellent biocompatibility with low immunogenicity [18]. In addition, the porous structure guides inward proliferation and the migration of fibroblasts and endothelial cells, which promotes granulation tissue and angiogenesis for WH with less scarring [21,22].

It is known that functional surface coatings mediate the cell-surface interaction between tissues and biomaterials, which provide alternative strategies for the improvement of bioactivities [25,26].

### 3.6. Outcome Evaluation Methods and Adverse Effects

Endpoint evaluation methods included biopsies, photographic evaluation, global photographs, physician global assessment score, and patient global assessment score. The satisfaction surveys and scales were taken from the perspective of patients, and other observers were also used to evaluate the efficacy of RS in some of the recruited studies.

About 84% of the studies analyzed (60/72) showed effective outcomes in term of WH and an improvement of STV compared with the baseline, while a few pooled patients reported adverse effects, including mild pain at injection sites after 48 to 72 hours, which would resolve spontaneously on the fourth or fifth post-operation day.

### 3.7. Selected Studies Analyzed

De Angelis et al. [61] published a recent study in which 35 patients affected by chronic vascular ulcers with a mean area of 35.1 ± 31.8 cm^2^ were treated with a dermal substitute (DS) called Nevelia^®^, followed by an autologous dermal epidermal graft (DEG). Follow-up was performed at 7, 14, 21, and 28 days after the DS implant and 7, 14, 21, and 28 days after the DEG. At 28 days after the DEG, the mean values of the Manchester Scar Scale were 1.8 ± 0.7 for skin color, 1.6 ± 0.7 for skin contour, 1.7 ± 0.7 for distortion, and 1.7 ± 0.7 for skin texture, whereas skin was matte in 27 patients (77%) and shiny in the remaining eight cases (23%). Histological findings correlate with the clinical results showing regenerated skin with reactive epidermal hyperplasia and dermal granulation tissue after two weeks (T1), and after three weeks (T2), a re-epithelialization and formed new tissue architecture analogous to normal skin physiology.

De Angelis et al. [62] reported on an in vitro and in vivo evaluation of a bio-functionalized scaffold composed of PRP and hyaluronic acid (HA) used in 182 patients affected by chronic ulcers (diabetic and vascular), comparing the results with a control group of 182 patients treated with traditional dressings (HA alone). After 30 days, the patients who had undergone the combined treatment (PRP and HA), showed 96.8% ± 1.5% re-epithelialization, as compared to 78.4% ± 4.4% in the control group (HA only). Within 80 days, they had 98.4% ± 1.3% re-epithelialization as compared to 87.8% ± 4.1% in the control group (HA only; *p* < 0.05). PRP and HA treatment showed stronger regenerative potential in terms of epidermal proliferation and dermal renewal compared with HA alone.

Gentile et al. [27,28,29,30,31,33,34,35,37,39,40,44,48,49,50,51] published several articles reporting the clinical and microscopic outcomes of autologous fat graft enriched with AD-MSCs/stromal vascular fraction cells (SVF) or with PRP in burn or post-traumatic scars [31,37,38,39], breast reconstruction [28,30,33,35,36], and breast augmentation [51], analyzing the different methods of cells isolation [29,50] and comparing the results with traditional procedures [46]. In patients who received SVF/AD-MSCs or PRP-enriched fat graft, the percentage of soft tissue volume maintenance was 63% and 69%, respectively, compared with the control group (39%), treated with fat graft not-enriched, after 1 year. MRI and ultrasound examinations showed lower fat reabsorption in the SVF and PRP groups, as confirmed also by Fiaschetti et al. [32].

Several studies analyzed showed that AD-MSCs modulate cancer growth [27,36] and promote wound repair [27]. The resorption percentage of fat grafting in obesity phenotypes [34], the role of SVF in different fat graft preparation procedures as nano-fat [39], the biomolecular basis of AD-MSC differentiation [43] in three-dimensional collagen scaffolds [41] and their apoptosis [42], mechanisms promoting the clinical fat graft maintenance [44,45], and finally, the possibility of using an allogeneic fat graft [49] were also analyzed.

Cervelli et al. [47] showed that CSW treated with e-SVF healed better than those treated with hyaluronic acid. In fact, after 9.7 weeks, patients treated with e-SVF underwent 97.9% ± 1.5% re-epithelialization compared to 87.8% ± 4.4% of the first control group (only hyaluronic acid; *p* < 0.05). Patients treated with PRP and fat grafting also showed an improvement in re-epithelialization; in fact, after 9.7 weeks, they underwent a 97.8% ± 1.5% re-epithelialization compared to 89.1% ± 3.8% of the second control group (only PRP; *p* < 0.05). As reported, e-SVF and PRP mixed with fat grafting were the two treatments evidencing improvement in the healing of patients affected by CSWs and post-traumatic extremity ulcers.

The role of PRP in wound healing and hair regrowth has been recently reported [52], describing the positive effects of autologous GFs contained in activated and not-activated PRP [53,54,56] in androgenetic alopecia comparing, the results with a micro-needling technique [55], low-level LED therapy [55], micrografting [57], Minoxidil^®^, Finasteride^®^, and adult stem cell-based therapy [58].

The details of the studies analyzed, describing their advantages and disadvantages and comparisons with the strategies available, have been summarized in Table 1.

## 4. Discussion

The use of autologous fat grafting and related AD-MSCs have become popular surgical procedures during the past 10 years, in the treatment of STDs, CSW, scars, and chronic ulcers [27,28,29,30,31,32,33,34,35,36,37,38,39,40,41,42,43,44,45,46,47,48,49,50,51].

As in every surgical procedure, the success of fat grafting is dependent upon many factors: the techniques and instruments used to harvest the fat tissue, the fat tissue processing, the volumes of fat implantation, the recipient sites to be implanted, the levels of placement, and even the individual patient. Because of this variability and perhaps because of other factors that are not yet understood, the results of fat grafting with some techniques, in some patients, and in some areas, can be unpredictable. A standard procedure has not been adopted by all practitioners. There is no agreement as to the best way of processing the fat to ensure the maximal take and viability of the graft [27,28,29,30,31,32,33,34,35,36,37,38,39,40,41,42,43,44,45,46,47,48,49,50,51]. Successful, three-dimensional sculpting requires meticulous planning and optimizing the harvesting, storage, and transplantation of adipose tissue. There is an unpredictable degree of resorption of the transplanted fat and repeated treatment sessions are usually needed in order to achieve the final result. The average tissue loss because of reabsorption after injection in the buttock varies between 24% and 84% [27,28,29,30,31,32,33,34,35,36,37,38,39,40,41,42,43,44,45,46,47,48,49,50,51]. The concept of refining techniques for liposuction and fat injection, according to individual anatomical zones, is essential to the evolution of the procedure [27,28,29,30,31,32,33,34,35,36,37,38,39,40,41,42,43,44,45,46,47,48,49,50,51].

Recently, an increasing number of papers have been reported on the use of fat grafting enhanced with stromal vascular fraction cells (SVFs), including breast reconstruction [28,29,30,32,33,35], scars [37,38,39,40,44], ulcers [31,41,47,49], and breast augmentation [51].

AD-MSCs may be identified in the mixed cell population, referred to as SVFs [37,38,39,40,47,49,51]. SVFs containing AD-MSCs might increase fat graft maintenance by improving vascularity and via the secretion of GFs that increase fat survival with the final aim of improving STV.

Several investigators have published articles using fat grafting enhanced with SVFs and AD-MSCs, called by different commercial names, with favorable results employing different procedures of cell isolation [28,29,30,32,33,35,37,38,39,40,41,47,49,51]. Additionally, several studies have been published on the use of centrifuged fat grafting enriched with PRP in Regenerative Plastic Surgery (RPS) [31,32,33,37,38,40,44,45] as RS, compared with traditional procedures [46].

Full-thickness skin defects caused by severe trauma, extensive burns, diabetes, and other reasons are common clinical disorders; their healing is a dynamic and multi-stage process that includes inflammation, angiogenesis, matrix deposition, and cell recruitment [63,64]. Additionally, there are multiple kinds of cells involved, including keratinocytes, endothelial cells, fibroblasts, inflammatory cells, and so forth. [65]. Generally speaking, the conventional treatment is to debride the wound and then perform skin grafting or skin flap transplantation after the wound granulation tissue grows well [2]. Compared with skin flap transplantation, skin grafts have the distinctive advantages of less damage to the donor site and easy operation [4]. However, due to the lack of a sufficient dermal matrix, skin grafts are easily hindered by uncontrollable scar hyperplasia, lower mechanical resistance, and so forth in the later phase [66]. The development and application of skin tissue engineering may improve skin graft treatment to a certain extent. PRP has been reported to be a stocking source for various GFs (e.g., PDGF, IGF-1, VEGF, FGF-2, etc.), which stimulate neo-angiogenetic vascularization and various activities of fibroblasts [62]. VEGF is accepted as the principal stimulatory factor of angiogenesis. PDGF, capable of enlarging blood vessels and forming mature vessels, is a powerful chemoattractant for fibroblasts and smooth muscle cells. Nevertheless, the application of PRP is currently limited by the short half-life period and the burst release (causing low concentration of GFs in situ) [12]. It is necessary to find solutions to improve the clinical application of PRP.

Artificial dermis is a commercially available bi-layer scaffold (including the silicone layer and the collagen-based layer) that has been widely applied in various full-thickness skin defects. The porous structure and suitable pore size of the lower layer (that is, the collagen sponge scaffold (CSS), termed as a dermal regenerate template (DRT)) promote cell adhesion and diffusion, and the connection of pores plays a key role in nutrient transportation and vascularization, significantly reducing contracture and scar formation [67]. Currently, a two-step surgical procedure is the universal criteria in the application of artificial dermis. Briefly, wounds are implanted with bi-layer artificial dermis, following thorough wound debridement in the first stage, and after two to three weeks of the DRT’s vascularization, skin grafts are performed to cover the wound bed in the second stage [68,69]. Undoubtedly, the need for two surgical procedures is not only indeed frustrating and inconvenient, but also increases the hospital stay and the risk of infection [70]. Therefore, one-step transplantation (i.e., DRT implantation and skin transplantation are performed in one surgical procedure) implies an attractive clinical prospect. However, the current DRT of artificial dermis commercially available by means of the one-step strategy will counter-intentionally form a barrier between the wound bed and the grafted skin, leading to graft failure [71].

Previous studies have suggested that PRP has positive effects on the implantation of artificial dermis. Formigli et al. [10] reported that artificial dermis combined with PRP demonstrated an improved overall restoration of tissue functions. On the other hand, Harrison et al. [72] explored the use of collagen as a platelet activator in PRP, proving the sustained release effect for growth factors from PRP with collagen. The main challenge of this strategy was understanding how to promote the cells’ adhesion, and proliferation, and vascularization into the scaffolds in the short-term after surgery, thus achieving sustained and efficient tissue regeneration with enough vascularization to enable the skin grafts with desired survival.

## 5. Concluding Remarks

In conclusion, this systematic review showed the efficacy of PRP, AD-MSCs, and biomaterials in the therapy of CSW, STDs, and skin defects. Given the current treatments differing in methodology and treatment technique, further studies are needed to define standardized protocols, and large-scale randomized trials still need to be conducted to confirm their efficacy. For these reasons, the authors invite all the audience to improve the level of publications in this field by focusing prevalently on EBM Level 1 studies.

## Figures and Tables

**Table 1:** Details of the studies analyzed compared with the strategies available, describing their advantages and disadvantages.

Strategies Analyzed	Advantages	Disadvantages	Strategies Available	Advantages	Disadvantages
Dermal substitutes (DS)	Granulation tissue and angiogenesis for wound healing with less scarring	Costs	Skin grafting or skin flap transplantation	Skin grafts offered less damage to the donor site and easy operation compared with skin flap transplantation	Skin grafts are easily hindered by uncontrollable scar hyperplasia and lower mechanical resistance, due to the lack of sufficient dermal matrix
	Faster wound healing compared with control groups (hyaluronic acid, skin grafts, advanced dressing, etc.)	Need to be followed by autologous dermal epidermal graft (two-step surgical procedure)			
	Re-epithelialization and formed new tissue architecture analogous to normal skin physiology				
Platelet-Rich Plasma (PRP)	Autologous treatment	Need to have authorization by the transfusion service	Hyaluronic acid, and/or advanced dressing	Lower cost compared with PRP	Lower regenerative potential in terms of epidermal proliferation and dermal renewal compared with PRP
	Faster wound healing compared with control groups (hyaluronic acid, skin grafts, advanced dressing etc.)	Need to repeat the treatment more times			
	Mini-invasive procedure				
	Improving neo-angiogenetic vascularization and various activities of fibroblasts				
Adipose-derived Mesenchymal Stem Cells (AD-MSCs)	Autologous treatment	Need to repeat the treatment more times	Fat grafting or hyaluronic acid	Fat grafting is considered an autologous treatment	Hyaluronic acid is synthetic dressing
	Faster wound healing compared with control groups (hyaluronic acid, skin grafts, advanced dressing etc.)	Not an easy procedure, which requires consolidated clinical practice		Hyaluronic acid application is an easy and fast procedure	Fat grafting presents a lower soft tissue volume maintenance compared with enriched AD-MSCs fat grafting
	Better soft tissue volume maintenance when they have been used to enrich fat graft, compared with fat graft alone	Costs			
	Improving vascularity via the secretion of growth factors				
